# Expression of S100A9 and KL-6 in common interstitial lung diseases

**DOI:** 10.1097/MD.0000000000029198

**Published:** 2022-05-06

**Authors:** Li Lin, Yabin Zhao, Zhenhua Li, Yun Li, Wei Wang, Jian Kang, Qiuyue Wang

**Affiliations:** a Department of Pulmonary and Critical Care Medicine, Institute of Respiratory Disease, The First Hospital of China Medical University, Shenyang, China; b Department of Geriatric Respiratory, The First Hospital of Kunming Medical University, Kunming, China.

**Keywords:** bronchoalveolar lavage fluid, interstitial lung disease, Klebs von den Lungen-6, S100 calcium binding protein A9

## Abstract

By evaluating S100 calcium binding protein A9 (S100A9) and Klebs von den Lungen-6 (KL-6) expression in patients with 4 common interstitial lung diseases (ILDs), we aimed to investigate whether S100A9 or KL-6 can be of any value in the differential diagnosis of these ILDs and simultaneously signal the disease progression.

We collected the data of patients diagnosed with the 4 ILDs and underwent fiber-optic bronchoscopy and BAL in the First Affiliated Hospital, China Medical University from January 2012 to December 2020. The data related to BGA, C-reactive protein, pulmonary function test, total number and fraction of cells, T lymphocyte subsets in bronchoalveolar lavage fluid (BALF), and the expression of S100A9 and KL-6 in BALF and serum were collected. We analyzed, whether S100A9 or KL-6 could serve as a biomarker for differential diagnosis between the 4 common ILDs; whether the levels of S100A9 and KL-6 correlated with each other; whether they were correlated with other clinical parameters and disease severity.

This study included 98 patients, 37 patients with idiopathic pulmonary fibrosis (IPF), 12 with hypersensitivity pneumonitis, 13 with connective tissue disease-associated ILD, and 36 with sarcoidosis (SAR): stage I (18), stage II (9), stage III (5), and stage IV (4). The expression of KL-6 in BALF was significantly higher in IPF patients than other 3 groups (all *P*-value < .05). However, there was no significant difference in the levels of S100A9 in BALF and serum between the 4 groups (*P*-value > .05). The levels of S100A9 in BALF of IPF patients was positively and significantly correlated with KL-6 expression and the percentage of neutrophils in BALF (*P*-value < .05). Along with the stage increase of SAR patients, the level of S100A9 in BALF gradually increased, which was negatively and significantly correlated with the forced vital capacity/predicted, carbon monoxide diffusing capacity/predicted%, and PaO_2_ (all *P*-value < .05).

The expression of KL-6 in BALF can be used as a biomarker to differentiate IPF from the other 3 common ILDs. While, this was not the case with expression of S100A9 in BALF and serum. However, the expression S100A9 in BALF is useful to indicate the progression of SAR. Thus, simultaneous measurement of KL-6 and S100A9 levels in BALF makes more sense in differential diagnosing of the 4 common ILDS.

## 1 Introduction

Interstitial lung diseases (ILDs) comprise a group of heterogeneous diseases involving lung parenchyma, characterized by fibroblasts overgrowth and extracellular matrix deposition, resulting in respiratory dysfunction, that is often fatal.^[1,2]^ Most common diseases in this group include idiopathic pulmonary fibrosis (IPF), connective tissue disease-associated interstitial lung disease (CTD-ILD), hypersensitivity pneumonitis (HP), cryptogenic organizing pneumonia, sarcoidosis (SAR), etc. Each of these diseases have their own characteristics, but share common clinical, radiological, and pathological features.^[3]^ Thus, the differentiation can be challenging, even after multidisciplinary discussion among the experienced experts.^[4]^ Moreover, the therapeutic outcomes and prognosis of these diseases differ drastically. For example, the prognosis in some ILDs such as CTD-ILD, HP, cryptogenic organizing pneumonia, and SAR are better than IPF, and the response to immunosuppressive therapy among these patients is good. In this scenario, having specific markers which help in differentiation or evaluation of progression could be of great value. Among various investigated factors, including Klebs von den Lungen-6 (KL-6), surfactant protein A, surfactant protein D, matrix metallopeptidase 7, CCR-like protein 18, CCR-like protein 13, and S100 calcium binding protein A9 (S100A9), which could be used for diagnosis, risk-stratification, prediction, and monitoring of treatment response of common ILDs,^[5–7]^ S100A9 and KL-6 are thought to be the most promising markers in the differential diagnosis and evaluation of prognosis of ILDs.^[7,8]^ However, their role as biomarkers of ILDs are limited and not unanimous.^[8–16]^

KL-6, a glycoprotein, is encoded by the MUC1 gene and expressed on the outer surface of injured and regenerating alveolar epithelial type II cells and airway epithelial cells. The surface expression of KL-6 is induced during the regeneration process of type II pneumocytes, resulting in an increased concentration in bronchoalveolar lavage fluid (BALF).^[17]^ Moreover, the destruction of the air–blood barrier alters the lung permeability, leading to higher serum concentrations of KL-6. Several previous studies have reported a strong correlation between BALF and serum concentrations of KL-6.^[18]^ Expression of KL-6 in serum and BALF has been reported to be associated with the progression and prognosis of several ILDs, and has been used to evaluate the efficacy of drugs for several ILDs; however, the results of some studies are inconsistent.^[17,19–22]^ Moreover, the views of certain researchers are contradictory,^[23,24]^ and thus, needs to be explored further.

S100A9, also known as myeloid-related protein 14 or calgranulin B, belongs to a family of calcium-binding protein S100, and is mainly expressed in neutrophils. Moreover, it could also be expressed on vascular endothelial cells and mature macrophages, under the influence of various chronic inflammatory factors.^[25]^ It has proinflammatory, profibrotic, and immunomodulatory properties.^[26–28]^ It can be specifically secreted in inflammatory lesions,^[28,29]^ is reported to be a potent therapeutic target for IPF,^[30]^ and may be a promising candidate biomarker to discriminate between IPF and other fibrotic interstitial pneumonias.^[8,31,32]^ However, this is not backed by the currently available evidences.

Based on the analyses above, in this study, we quantified the expression of S100A9 and KL-6 in serum and BALF of the 4 common ILDs, investigated whether S100A9 and/or KL-6 could serve as biomarkers for differential diagnosis between the 4 common ILDs.

## 2 Research subjects

### 2.1 Inclusion criteria

In this prospective study, we included inpatients aged between 18 and 80, admitted from January 2012 to December 2020, meeting the diagnostic criteria of the 4 common ILDs. All patients underwent fiber-optic bronchoscopy and bronchial alveolar lavage in the Department of Respiratory and Critical Care Medicine, First Affiliated Hospital, China Medical University. The ethics committee of the First Hospital of China Medical University approved this study. This study strictly adhered to the guidelines of the Declaration of Helsinki.

The diagnostic criteria for IPF was strictly in accordance with the consensus of the American Thoracic Society (ATS) and the European Respiratory Society Experts^[33]^; The diagnosis of SAR was strictly in accordance with the consensus of the ATS/ERS/WASOG committee statement on Sarcoidosis (1999)^[34]^; The diagnosis of CTD-ILDs were clinically classified as having rheumatoid arthritis, polymyositis and dermatomyositis, systemic sclerosis, or Sjögren syndrome, according to the criteria for respective diseases; and the diagnosis of HP was strictly in accordance with the consensus of the ATS/JRS/ALAT Clinical Practice Guideline.^[35–39]^

Pulmonary SAR was classified into 4 stages according to the chest computed tomography imaging, as per the previous reports.^[40]^

A multidisciplinary team including at least 2 pulmonologists, a radiologist, and a pathologist, all experts in ILDs, reviewed the data and confirmed the diagnosis. None of the patients enrolled in the study had ever received steroids, other immunosuppressants, or antifibrotic drugs.

### 2.2 Exclusion criteria

Patients younger than 18 or older than 80 years; not agreeing to participate in the study, or cannot sign the informed consent; pregnant or lactating women; severe cases unable to tolerate bronchoscopy examination; with concurrent infection; receiving immunosuppressant, glucocorticoid, or antifibrotic drugs; and those comorbid with other lung diseases, such as asthma, chronic obstructive pulmonary disease, bronchiectasis, tumor, silicosis, etc and with severe cardiovascular system, liver and kidney dysfunction, hematological diseases, and other autoimmune diseases were excluded from the study.

## 3 Experiment methods

Data related to demographic, clinical, and laboratory details included age, gender, smoking history, physical examination findings including vital sign, chest and abdomen, limbs, and nervous system examination were performed. Moreover, signs including clubbing and Velcro crackle were emphasized. Investigations included arterial blood analysis, peripheral blood cell counting, total cell counting and cytological classification, C-reactive protein, T lymphocyte subgroup in BALF, and liver kidney and cardiac function were performed in the Medical Laboratory Department, First Affiliated Hospital, China Medical University.

### 3.1 Patient's pulmonary function test (PFT) examination

PFT was performed using the lung function tester (Jaeger, Germany), and parameters such as forced vital capacity/predicted (FVC%), forced expiratory volume in 1 second/predicted, and carbon monoxide diffusing capacity/predicted (DLCO%) were recorded in all the patients. PFT was carried out by professional technicians from Institute of Respiratory Diseases, the First Affiliated Hospital, China Medical University.

### 3.2 Collection and preparation of BALF and serum samples

All the patients received pulmonary lavage of right middle lobe or left lingual lobe and the recovery rates of BALF specimens were greater than 35% and no blood were mixed in the lavage. In all the enrolled patients, 5 mL of fasting venous blood was withdrawn. Both the serum and BALF samples were placed in a heat preservation box and immediately sent to the laboratory for preparation. After the blood sample had been stationary for 1 to 2 hours, it was centrifuged at 4°C, at a rate of 3000 rpm for 10 minutes, and then the serum was separated and stored at −80°C prepared for subsequent experiments.

The BALFs were filtered through a double layered sterile gauze and placed in a plastic centrifuge tube and centrifuged at 4°C, at a rate of 1200 rpm for 10 minutes, following which the supernatant was separated. The supernatant was then poured into a sterile dialysis bag and immersed in 50% polyethylene glycol solution. After 24 hours, physiological saline (10-fold concentration) was added, and the concentrated solutions were frozen (−80°C) for storage and prepared for subsequent experiments.

### 3.3 Detection of S100A9 and KL-6 levels in serum and BALF

Enzyme-linked immunosorbent assay method was used. The kit was purchased from R&D Systems (Shanghai, China), and the concentrations of S100A9 (ng/mL) and KL-6 (U/mL) were calculated using a standard curve, in strict accordance with the instructions provided by the manufacturer.

### 3.4 Statistical analysis

Parametric and nonparametric statistics was applied in the present study. The continuous data was expressed as mean ± standard deviation. To evaluate the differences among groups, if the variance was even, then one-way ANOVA was used. While, if the variance was not even, then Games–Howell test was used. Correlation between the parameters was assessed by using Pearson or Spearman rank correlation for normally and non-normally distributed data, respectively. The absolute value of correlation coefficient (*r*) above 0.8, between 0.3 and 0.8, and below 0.3 denoted a strong, weak, and no correlation, respectively. The receiver operating characteristic (ROC) curves were used to evaluate the performance S100A9 and KL-6 in the diagnosis of IPF, and power analysis were performed. *P* < .05 was considered as statistically significant. Data analyses were performed using SPSS 20.0 and Graph Pad Prism 4.0 software.

## 4 Results

### 4.1 Demographic and clinical data (Table 1)

There were 37 subjects in IPF group, 12 in HP group, 13 in CTD-ILD group, and 36 in SAR group. In the IPF group, the proportion of males and mean age were significantly higher than those in the HP, CTD-ILD, and SAR group (*P*-value < .05). Moreover, the proportion of smokers in the IPF group were significantly higher than that in the SAR group (*P*-value < .05). However, there were no significant differences between the groups in terms of acropachy and Velcro crackle (all *P*-value > .05).

**Table 1:** Demographic and clinical characteristics of ILD patients (x ± SD).

Group	Subjects	Male (%)	Age	Smoking (%)	Acropachy (%)	Velcro (%)
IPF	37	35 (95)	61.73 ± 7.19	65	9 (24)	12 (20)
HP	12	6 (50)^∗^	48.00 ± 8.36^∗^	33	0 (0)	2 (17)
CTD-ILD	13	5 (38)^∗^	53.46 ± 11.08^∗^	31	1 (8)	2 (15)
SAR	36	15 (42)^∗^	48.33 ± 10.63^∗^	9^∗^	4 (11)	10 (28)

CTD-ILD = connective tissue disease-associated interstitial lung disease, HP = hypersensitivity pneumonitis, ILD = interstitial lung disease, IPF = idiopathic pulmonary fibrosis, SAR = sarcoidosis.

∗ Compare with IPF group *P*-value < .05.

### 4.2 Pulmonary function test and BGA (Tables 2 and 3)

There was no significant difference between all the 4 groups in terms of forced expiratory volume in 1 second/predicted (all *P*-value > .05). However, the FVC%, DLCO%, and PaO_2_ levels in the IPF group were significantly lower than those in the SAR group (*P*-value < .05). Similarly, the DLCO% and PaO_2_ levels in the CTD-ILD group were significantly lower than those in the SAR group (*P*-value < .05) (see Table 2). However, as the disease progressed, separate analysis of PFT and PaO_2_ among the SAR patients revealed a downward trend. And, specifically, DLCO% and PaO_2_ level were significantly lower in SAR stage III and stage IV group than the SAR stage I group (both *P*-value < .05) (see Table 3).

**Table 2:** PFT and PaO_2_ of patients in the 4 groups (x ± SD).

Group	FEV1%	FVC%	DLCO%	PaO_2_ (mm Hg)
IPF	77.08 ± 13.31	69.02 ± 13.68	58.56 ± 23.59	75.06 ± 11.92
HP	75.85 ± 15.17	72.63 ± 15.01	63.85 ± 27.36	77.08 ± 16.98
CTD-ILD	76.84 ± 12.73	74.02 ± 11.91	53.31 ± 16.06^†^	74.56 ± 6.17^†^
SAR	81.04 ± 10.03	76.86 ± 10.22^∗^	72.47 ± 15.76^∗^	83.59 ± 11.11^∗^

CTD-ILD = connective tissue disease-associated interstitial lung disease, DLCO = carbon monoxide diffusing capacity, FEV1 = forced expiratory volume in 1 s, FVC = forced vital capacity, HP = hypersensitivity pneumonitis, IPF = idiopathic pulmonary fibrosis, PFT = pulmonary function test, SAR = sarcoidosis.

∗ Compare with IPF group *P*-value < .05.

† Compare with SAR group *P*-value < .05.

**Table 3:** PFT and PaO_2_ of patients in different stages of sarcoidosis (x ± SD).

Group	FEV1%	FVC%	DLCO%	PaO_2_ (mm Hg)
SAR stage I	83.21 ± 9.18	80.36 ± 10.82	80.41 ± 10.78	90.35 ± 8.21
SAR stage II	80.41 ± 12.96	74.74 ± 9.43	70.51 ± 13.04	84.33 ± 3.64
SAR stage III	78.22 ± 9.55	73.20 ± 7.53	64.20 ± 20.29^∗^	70.38 ± 6.91^∗^
SAR stage IV	76.20 ± 7.27	70.45 ± 8.83	51.50 ± 12.04^∗^	68.00 ± 8.29^∗^

DLCO = carbon monoxide diffusing capacity, FEV1 = forced expiratory volume in 1 s, FVC = forced vital capacity, PFT = pulmonary function test, SAR = sarcoidosis.

∗ Compare with SAR stage I group *P*-value < .05.

### 4.3 Total count and percentage of each cell fraction in BALF (Table 4)

The mean percentage of lymphocytes were significantly lower in IPF group than that in SAR group, while, the percentage of macrophages in BALF were significantly higher in IPF group than in SAR group (both *P*-value < .05). The CD4/CD8 ratio was significant higher in SAR group and lower in HP group than in IPF group (data were not shown, both *P*-value < .05). There was no significant difference between the 4 groups in terms of total mean cell counts, and the mean percentages of neutrophils and eosinophils in BALF (all *P*-value > .05).

**Table 4:** Total cell count and the percentage of each cell fraction in BALF of patients in the 4 groups (x ± SD).

Group	Total number (×10^5^/mL)	M (%)	N (%)	L (%)	E (%)
IPF	1.52 ± 1.05	77.27 ± 8.29	9.35 ± 7.14	12.89 ± 7.81	0.49 ± 0.77
HP	2.20 ± 1.78	65.00 ± 17.67	8.33 ± 3.39	26.08 ± 18.15	0.58 ± 1.00
CTD-ILD	1.73 ± 1.28	72.85 ± 18.50	8.77 ± 10.70	17.31 ± 15.75	1.08 ± 2.22
SAR	1.66 ± 1.22	62.83 ± 15.88^∗^	7.03 ± 4.12	29.86 ± 16.20^∗^	0.25 ± 0.73

BALF = bronchoalveolar lavage fluid, CTD-ILD = connective tissue disease-associated interstitial lung disease, E = eosinophil, HP = hypersensitivity pneumonitis, IPF = idiopathic pulmonary fibrosis, L = lymphocyte, M = macrophage, N = neutrophil, SAR = sarcoidosis.

∗ Compare with IPF group *P*-value < .05.

### 4.4 KL-6 expression in BALF and serum

**Figure 1:**
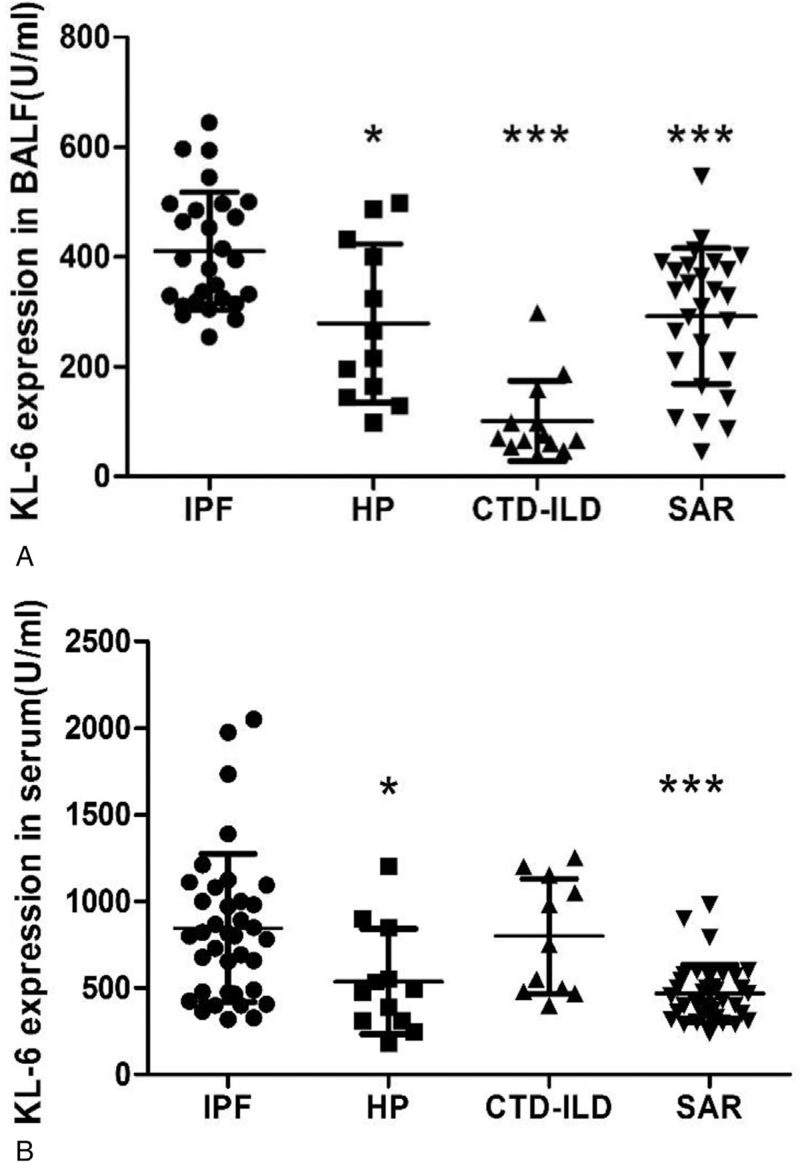
KL-6 expression in serum and BALF of ILD patients. (A) KL-6 expression in BALF; (B) KL-6 expression in serum. ∗Compared with IPF group *P*-value < .05; ∗∗∗Compared with IPF group *P*-value < .001. BALF = bronchoalveolar lavage fluid, CTD-ILD = connective tissue disease-associated interstitial lung disease, HP = hypersensitivity pneumonitis, ILD = interstitial lung disease, IPF = idiopathic pulmonary fibrosis, KL-6 = Klebs von den Lungen-6, SAR = sarcoidosis.

**Figure 2:**
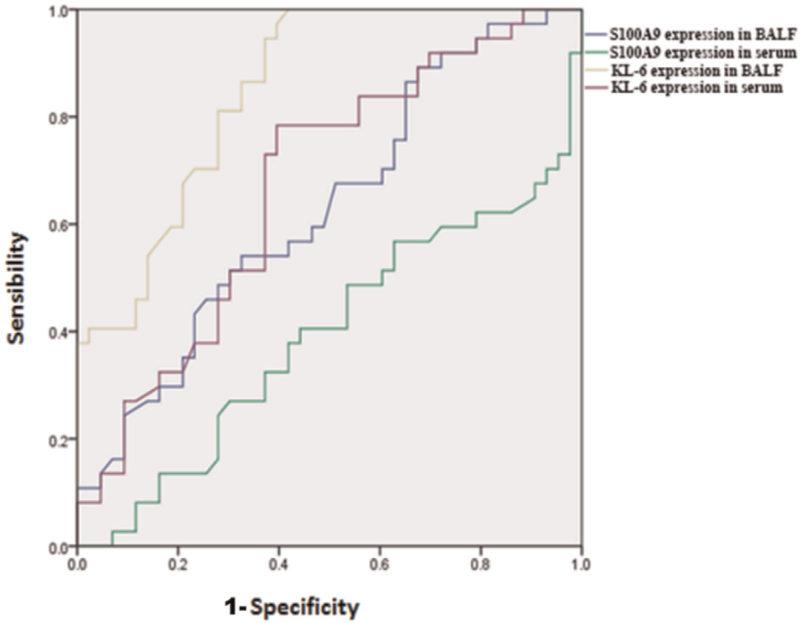
ROC curve. BALF = bronchoalveolar lavage fluid, KL-6 = Klebs von den Lungen-6, S100A9 = S100 calcium binding protein.

The expression of KL-6 in BALF of IPF patients was significantly higher than that of SAR, HP, and CTD-ILD patients (all *P*-value < .05). Similarly, the expression of KL-6 in serum of IPF patients was significantly higher than that of SAR, and HP patients (*P*-value < .05) (Fig. 1A, B). The ROC curve of BALF and serum KL-6 in the IPF group (Fig. 2), had the area under the curve of 0.89 and 0.73, with power analysis results 99% and 98%, respectively,^[41,42]^ indicating that KL-6 expression in BALF is a good candidate marker for the diagnosis of IPF (*P* < .05).

### 4.5 S100A9 levels in BALF and serum

**Figure 3:**
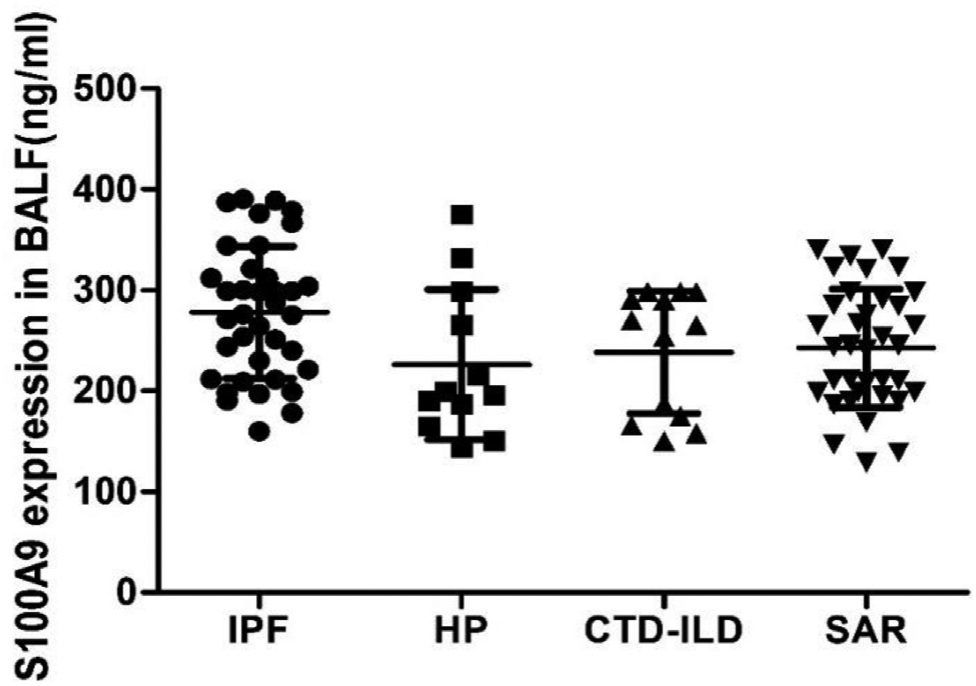
S100A9 expression in BALF of ILD patients. BALF = bronchoalveolar lavage fluid, CTD-ILD = connective tissue disease-associated interstitial lung disease, HP = hypersensitivity pneumonitis, ILD = interstitial lung disease, IPF = idiopathic pulmonary fibrosis, S100A9 = S100 calcium binding protein, SAR = sarcoidosis.

**Figure 4:**
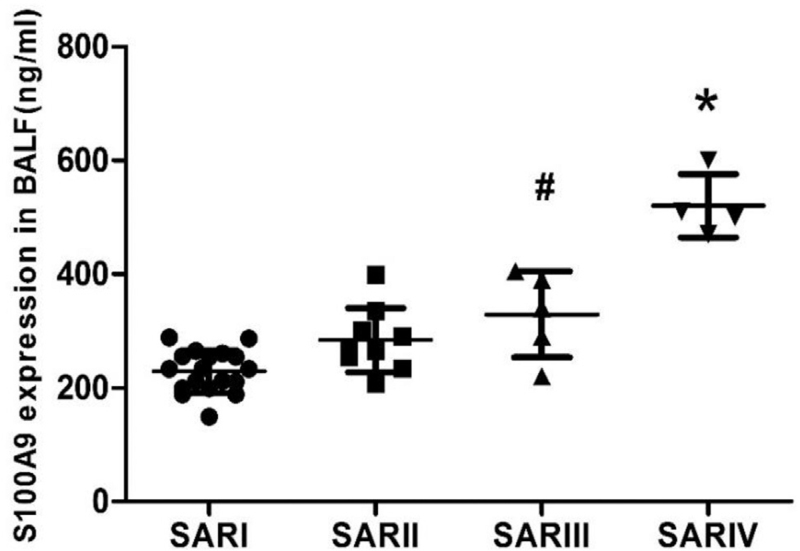
S100A9 expression in BALF of SAR patients. #Compared with SAR stage I group *P*-value < .05, ∗Compared with SAR stage I group, SAR stage II, and SAR stage III group *P*-value < .05. BALF = bronchoalveolar lavage fluid, S100A9 = S100 calcium binding protein, SAR = sarcoidosis.

Although, it seems that the level of S100A9 in BALF of IPF patients was higher than that of other groups, there was no statistically significant difference between all the groups (all *P*-value > .05) (Fig. 3). Moreover, as the SAR progressed, the level of S100A9 in BALF gradually increased and SAR III group had a higher mean value of S100A9 in BALF, as compared to SAR I group. Moreover, SAR IV group had a significant higher mean value of S100A9 in BALF, as compared to SAR I, SAR II, and SAR III groups (all *P*-value < .05) (Fig. 4). There was no difference between the 4 groups, as well as between the 4 stages of SAR in terms of S100A9 expression in serum. The ROC curve of BALF and serum S100A9 levels in the IPF group (Fig. 2), had the area under the curve of 0.67 and 0.44 (both *P* > .05), respectively indicating that S100A9 expression in BALF or serum could not be used as a reliable marker for the diagnosis of IPF in this study.

### 4.6 Correlation between S100A9 in BALF and lung function and other clinical indicators

**Figure 5:**
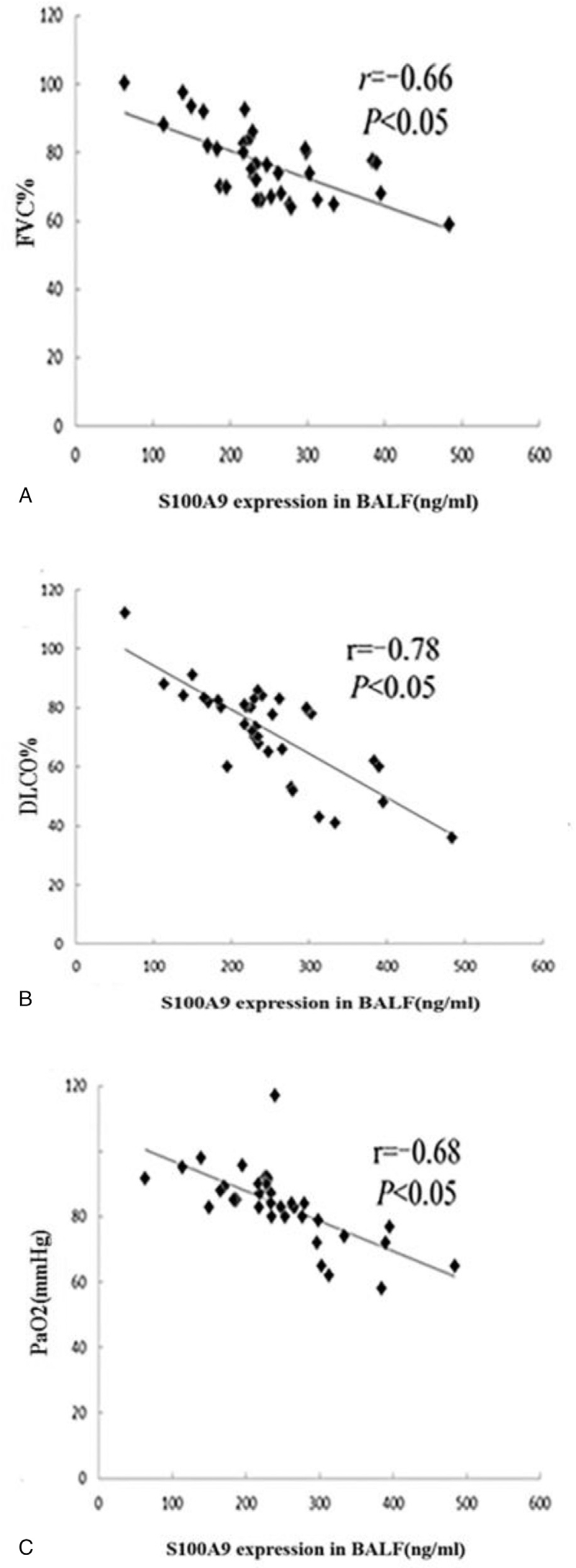
Correlation between S100A9 level in BALF and FVC% (A), DLCO% (B), PaO_2_ (C) in SAR patients. BALF = bronchoalveolar lavage fluid, DLCO = carbon monoxide diffusing capacity, FVC = forced vital capacity, S100A9 = S100 calcium binding protein, SAR = sarcoidosis.

Correlation analysis revealed that the level of S100A9 in BALF of IPF patients was positively and significantly correlated with KL-6 (*r* = 0.35, *P*-value < .01) and neutrophil count (N%, *r* = 0.62, *P*-value < .01) in BALF. However, there was no significant correlation with other clinical indicators (Table 5). Moreover, the levels of S100A9 in BALF of SAR patients were negatively and significantly correlated with FVC% (*r* = −0.66), DLCO% (*r* = −0.78), and PaO_2_ (*r* = −0.68) (all *P*-value < .05) (Fig. 5).

**Table 5:** Correlation between S100A9 in BALF and serum of IPF patients and other clinical indicators.

	S100A9 in BALF (ng/mL)		S100A9 in serum (ng/mL)	
	*r*	*P*	*r*	*P*
Age	−0.16	.36	0.17	.31
KL-6 in serum	0.15	.18	−0.13	.27
KL-6 in BALF	0.35	<.01^∗^	−0.13	.25
FVC%	−0.28	.09	−0.12	.47
DLCO%	−0.11	.50	0.05	.76
CRP	0.17	.30	−0.12	.49
PaO_2_	0.06	.73	−0.15	.38
M%	−0.25	.14	−0.08	.63
N%	0.62	<.01^∗^	0.04	.80
L%	−0.32	.54	−0.18	.28
E%	−0.04	.82	−0.03	.88
CD4/CD8	−0.02	.91	−0.12	.50

BALF = bronchoalveolar lavage fluid, CRP = C-reactive protein, DLCO = carbon monoxide diffusing capacity, E = eosinophil, FVC = forced vital capacity, IPF = idiopathic pulmonary fibrosis, KL-6 = Klebs von den Lungen-6, L = lymphocyte, M = macrophage, N = neutrophil, S100A9 = S100 calcium binding protein A9.

## 5 Discussion

ILD comprise a large group of parenchymal pulmonary disorders, thus, early and accurate diagnosis can be challenging. Moreover, it is difficult to predict the disease progression. Thus, tracking the behavior of the disease is crucial.^[43]^ A proportion of ILD patients may develop a progressive-fibrosing phenotype, with fast worsening of respiratory symptoms, decline in lung function, limited response to immunomodulatory therapies, lower quality of life, and potentially early death.^[43]^ The differential diagnosis of common ILDs requires a combination of medical history, laboratory assessment, radiological, and sometimes pathological examination.^[44,45]^ Moreover, the differentiation of IPF from other common ILDs is even more difficult.^[46]^ The clinical manifestations and radiological findings of some fibrotic ILDs (such as chronic HP, CTD-ILD, and sometimes SAR, etc) can be similar to IPF, but their prognosis differs drastically. Thus, in this study, we focused on common ILDs, including IPF, HP, CTD-ILD, and SAR which are difficult to diagnose and differentiate from progressive-fibrosing phenotype, and we aimed to investigate whether S100A9 and/or KL-6 could serve as a biomarker for differentiating and/or indicating the progression for these common ILDs.

A number of biomarkers have been explored to help in the differential diagnosis of common ILDs,^[47–51]^ among them, KL-6 is a most promising.^[8,19,31,32,52–55]^ KL-6 has also been reported to be associated with the progression of IPF and have been used for evaluating the efficacy of drugs targeting IPF, although its role is disputed.^[19,22,47,56–62]^

KL-6 is a mucin-1 glycoprotein of the genus Cluster9 encoded by MUC1 gene, mainly secreted by proliferating, reproducing, or damaged alveolar type II epithelial cells. Serum KL-6 levels can be used as an indicator of alveolar epithelial cell destruction and regeneration. KL-6 is also a cell–surface barrier and may have a defensive function associated with the damage to the alveolar epithelial barrier molecule. Since KL-6 is a mucin rather than a surfactant, overexpression of KL-6 may limit alveolar ventilation and ventilation functions, thus, affecting the pulmonary retention. In addition, it is a potential proliferative and antiapoptotic factor of lung fibroblasts and may be responsible for initiation and proliferation of fibrosis.^[63]^ Its level may change dynamically with disease progression.^[64]^

Data related to S100A9 in ILDs is scarce; however, available evidences suggest that S100A9 may be involved in the pathogenesis of IPF.^[8]^ Moreover, serum levels of S100A9 are reported to be elevated in patients with rheumatoid arthritis, systemic lupus erythematosus, scleroderma, vasculitis, lung cancer, etc.^[12,65–68,16]^ Some studies have demonstrated that it can promote atherosclerosis.^[69]^ S100A9 is associated with neutrophils in the blood and constitutes approximately 45% of neutrophilic cytoplasmic proteins.^[70]^ Its function is complex and includes both intracellular and extracellular functions. Intracellularly, it promotes inflammatory responses and upregulates inflammatory mediators. Extracellularly, some of its functions are exerted mainly through toll-like receptor 4 on cell surface or RAGE (late glycosylation end products) receptor binding, and is involved in cell signal transduction.^[71]^

S100A9 is also implicated in the pathogenesis of SAR,^[72]^ and is highly expressed in granulomas of SAR patients.^[31,73]^ Some studies have suggested that S100A9 has a role in differentiating IPF and SAR from other common pulmonary diseases. However, some studies have reported contrary findings, data in the literature regarding SAR are quite controversial. Moreover, such studies have not involved Chinese population.

In this study, the proportion of males and age of the patients in IPF group were significantly higher than those in HP, CTD-ILD, and SAR groups, thereby, demonstrating that IPF more commonly affects older men, and this finding is consistent with those cited in literature.^[49]^ The FVC%, DLCO%, and PaO_2_ levels of IPF patients in this study were lower than SAR group, which means IPF patients suffer from lower blood oxygen, poorer lung ventilation function and diffusion capacity than SAR patients. There was no significant difference between the 4 groups in terms of the percentage of neutrophils in BALF in current study, which is inconsistent with previous studies.^[74,75]^ This may be due to the relatively stable condition of the enrolled patients and the relatively small sample size of this study.

We found that the expression of KL-6 in BALF was higher in IPF group than other 3 groups and the KL-6 expression in serum was higher in IPF group than HP and SAR groups. Thus, levels of KL-6 in BALF might be used as an important biomarker to diagnose IPF. However, at present, there is lack of studies with large sample size evaluating the KL-6 level in ILD patients in China, and the role of KL-6 in the diagnosis, monitoring, and determining the disease activity in ILD patients, needs to be further clarified.

The levels of S100A9 in BALF were not significantly higher in IPF group than HP, CTD-ILD, and SAR groups. These results are not consistent with the previous finding.^[15]^ This may be related, firstly, to the relatively mild nature of disease in patients of IPF group in this study, and secondly, the small sample size in this study. It has been demonstrated that the levels of S100A9 were significantly elevated in BALF of patients with severe IPF. Moreover, there was no statistically significant difference between all the 4 groups in terms of the S100A9 levels in serum, and this is consistent with previous findings. The results of this study revealed that the levels of S100A9 in BALF of IPF patients were positively correlated with the percentage of neutrophils in BALF, thereby, suggesting that S100A9 may be associated with disease activity of IPF, which could explain absence of significant difference in S100A9 levels between the groups.

Some of the available studies have reported that the levels of S100A9 are significantly elevated in BALF of patients with pulmonary involvement in systemic sclerosis.^[76–79]^ However, other studies have demonstrated that the levels of S100A9 in BALF of patients with pulmonary involvement in systemic sclerosis did not differ from healthy controls. Therefore, whether levels of S100A9 in BALF can be used to distinguish between ILDs needs further investigation.

Pulmonary SAR is a common noncaseous granulomatous interstitial pulmonary inflammatory disease.^[79]^ We found that with the progression of SAR, DLCO% and PaO_2_ demonstrated a descending trend, and the levels of S100A9 in BALF gradually increased. Correlation analysis also revealed that S100A9 in BALF of SAR patients was negatively correlated with FVC%, DLCO%, and PaO_2_, thereby, suggesting that the level of S100A9 in BALF can reflect the severity of lung involvement in patients with SAR.

Though, our study reports some novel findings, it has certain limitations. Firstly, this study included patients from a single center and the sample size was small. Secondly, due to unavailability, we did not have access to BALF findings of healthy people, so there was a lack of healthy controls, thereby limiting its diagnostic significance as a biomarker. Thirdly, the nature of the study resulted in selection bias, and the timing of exposure and disease onset was difficult to determine. Finally, this is a pragmatic study that lacks randomized allocation and has diagnostic limitations. Thus, large longitudinal studies are needed before the translational use of the potential biomarkers in clinical practice.

## 6 Conclusion

The expression of KL-6 in BALF may be used as a promising differentiating biomarker for IPF and other ILDs. S100A9 in BALF may use as a predictor of disease progression in SAR. It could not be ascertained whether S100A9 in BALF can be used for the differential diagnosis of IPF and whether it is related to the activity of IPF, thus, needs to be evaluated further. The difficulty and high cost of performing fiberoptic bronchoscopy together with limitations of both measures, suggests that combined measurement of KL-6 and S100A9 in BALF could be more helpful in the differential diagnosis and assessing the disease activity of IPF and SAR.

## Author contributions

WQY, WW and KJ contributed to experimental design and statistical analysis; LL, ZYB, LZH and YL contributed to data collection and document writing.

**Conceptualization:** Jian Kang, Li Lin, Qiuyue Wang, Qiuyue Wang, Wei Wang, Yabin Zhao, Yun Li, Zhenhua Li.

**Data curation:** Qiuyue Wang.

**Formal analysis:** Li Lin, Qiuyue Wang.

**Funding acquisition:** Qiuyue Wang, Wei Wang.

**Investigation:** Li Lin, Qiuyue Wang, Yun Li.

**Methodology:** Jian Kang, Li Lin, Qiuyue Wang, Yabin Zhao.

**Project administration:** Qiuyue Wang, Zhenhua Li.

**Resources:** Qiuyue Wang, Yun Li.

**Software:** Li Lin, Qiuyue Wang.

**Supervision:** Qiuyue Wang.

**Validation:** Qiuyue Wang, Yun Li.

**Visualization:** Qiuyue Wang.

**Writing – original draft:** Li Lin, Qiuyue Wang.

**Writing – review & editing:** Jian Kang, Li Lin, Qiuyue Wang, Wei Wang.
